# Sperm performance of coastal northern pike (*Esox lucius L.*) from the Baltic Sea shows no impairment between freshwater and brackish conditions

**DOI:** 10.1093/conphys/coag022

**Published:** 2026-04-16

**Authors:** Amanda Viving, Caroline Ek, Martin Ogonowski, Joacim Näslund, Elin Dahlgren, John L Fitzpatrick, Léa Daupagne

**Affiliations:** Department of Zoology, Stockholm University, Svante Arrhenius väg 18B, Stockholm 106 91, Sweden; Institute of Freshwater Research, Swedish University of Agricultural Sciences, Stångholmsvägen 2, Drottningholm 178 93, Sweden; Institute of Freshwater Research, Swedish University of Agricultural Sciences, Stångholmsvägen 2, Drottningholm 178 93, Sweden; Institute of Freshwater Research, Swedish University of Agricultural Sciences, Stångholmsvägen 2, Drottningholm 178 93, Sweden; Institute of Freshwater Research, Swedish University of Agricultural Sciences, Stångholmsvägen 2, Drottningholm 178 93, Sweden; Department of Zoology, Stockholm University, Svante Arrhenius väg 18B, Stockholm 106 91, Sweden; Department of Zoology, Stockholm University, Svante Arrhenius väg 18B, Stockholm 106 91, Sweden

**Keywords:** Baltic Sea, northern pike (*Esox lucius*), reproductive ecology, recruitment decline, salinity difference, sperm motility

## Abstract

Declining coastal populations of northern pike (*Esox lucius*) in the Baltic Sea have raised concerns about potential reproductive constraints, including reduced sperm quality linked to changing salinity regimes. This study tested whether sperm performance in coastally collected pike is impaired by activation in freshwater versus brackish water. Sperm velocity and motility were measured from adult males collected at two Baltic Sea locations (Sankt Anna, 2024; Hanöbukten, 2025) and activated in either deionized (0 ppt) or local brackish water (6–7 ppt). We found that sperm velocity and motility declined rapidly over time post-activation, but did not differ significantly between salinity treatments during the critical fertilization window (***≤***20 s). At later time points (25–45 s), sperm in brackish water maintained higher velocities and motilities than in deionized water, indicating greater longevity under local conditions. Overall, our results show that sperm function in coastal northern pike is not limited by salinity difference, suggesting that current recruitment declines are unlikely to result from impaired male gamete performance. Instead, reproductive limitations in Baltic pike populations may arise from later developmental stages or environmental pressures on egg and larval survival.

## Introduction

The northern pike (*Esox lucius* L., hereafter pike) is a large predatory species with a key role in top-down regulation of freshwater and brackish fish communities across the northern hemisphere (Craig, 2008; Donadi *et al*., 2017; Eklöf *et al*., 2020). Beyond its ecological importance, the species holds considerable commercial value and is a major target of recreational fishing (Arlinghaus *et al*., 2018; Carlén *et al*., 2019). In the Baltic Sea, however, many coastal pike populations have experienced significant declines over the last 50 years (Lehtonen, 1986; Nilsson *et al*., 2004; Bergström *et al*., 2022). Declines in both commercial and recreational catches, indicative of decreasing population sizes, have been reported throughout the region (Hentati-Sundberg, 2017), ranging from 12 to 100% depending on the location (Olsson *et al*., 2023). Historically, declines in pike populations have been attributed to recruitment failures, potentially caused by over-harvesting and increased predation by great cormorants (*Phalacrocorax carbo*) and grey seals (*Halichoerus grypus*) on adult stocks (Östman *et al*., 2013; Hansson *et al*., 2018; Bergström *et al*., 2022). Yet, a recent telemetry study found that 84% of tagged adult pike survived a 10-month period, suggesting that adult mortality may be relatively low in some coastal populations (Flink *et al*., 2023). This shifts attention towards early life stages, where recruitment failures may instead reflect reduced reproductive success or limited survival during early development.

**Figure 1:**
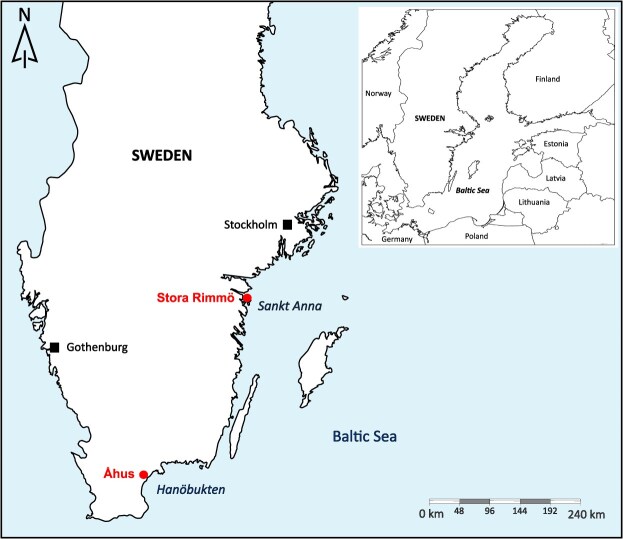
Study sites in the southern Swedish Baltic Sea, including the Sankt Anna Archipelago and Hanöbukten Bay. Red dots indicate sperm sampling locations near Stora Rimmö and Åhus.

While recent studies have highlighted the increase of three-spined stickleback (*Gasterosteus aculeatus*) predation on pike eggs and larvae (Nilsson *et al*., 2004; Bergström *et al*., 2015; Nilsson *et al*., 2019; Donadi *et al*., 2020), declining recruitment success may additionally reflect underlying reproductive issues. Supporting this view, a pike hatchery manager from the Sankt Anna Archipelago, Sweden (Fig. 1) has reported multiple reproductive issues in pike populations over recent years, including reduced fertilization and hatching success, despite standardized methods and infrastructure (K. Karlsson, personal communication, 2024). One potential factor that could reduce fertility and contribute to declining recruitment is reduced sperm quality. Although the implications of reduced male reproductive capacity for population-level processes remain incompletely understood, growing evidence suggests that paternal effects through variation in sperm quality constitute a key component of stock reproductive potential and may serve as a promising predictor of recruitment in fish stocks (Trippel, 2003; Kamler, 2005). In externally fertilizing fish such as pike, sperm velocity in particular is a critical determinant of fertilization success and is widely used as a proxy for male reproductive performance (Gage *et al*., 2004; Geßner *et al*., 2017). Sperm motility rapidly declines following activation, due to ATP depletion and increased membrane permeability (Christen *et al*., 1987; Billard *et al*., 1995; Perchec *et al*., 1995), making the time window shortly after sperm release particularly critical for successful fertilization (Billard *et al*., 1995; Linhart *et al*., 2008; Alavi *et al*., 2012). As sperm are directly exposed to the surrounding medium, subtle environmental or physiological factors that alter sperm activation and performance could strongly influence fertilization outcomes and, ultimately, recruitment success.

Among environmental factors, salinity plays a critical role in pike populations in the Baltic sea, which inhabit habitats spanning a broad salinity gradient. While pike typically is a freshwater resident species, in the Baltic Sea it displays various life-history strategies, including both migratory (anadromous) and brackish-water resident forms. Pike populations usually show strong site fidelity and limited dispersal (typically *<*5 km), contributing to local adaptation to specific spawning environments (Saulamo and Neuman, 2002; Laikre *et al*., 2005; Sunde *et al*., 2018). This includes local adaptation to a range of salinity regimes, with populations that spawn in coastal areas (i.e. brackish-resident pikes) often genetically distinct from their freshwater counterparts (Sunde *et al*., 2018; Nordahl *et al*., 2019). However, eutrophication and food web changes over the last decades has contributed to the deterioration of spawning habitat in brackish waters along the Baltic Sea coast (Lehtonen, 1986; Jansson and Dahlberg, 1999; Sandström and Karås, 2002). Such environmental changes could reduce the suitability of brackish spawning habitats for locally adapted pike populations, therefore increasing reliance on freshwater spawning areas, including ‘pike factories’ (i.e. restored or constructed coastal wetlands) designed to enhance recruitment. Previous work has shown that pike can exhibit flexible migration and habitat use behaviour (Roser *et al*., 2023; Rittweg *et al*., 2024), suggesting that some individuals may be capable of exploiting these alternative spawning habitats. Although the necessary condition for sperm motility activation in most fish is a change in the osmolality of the medium (Morisawa and Suzuki, 1980; Morisawa *et al*., 1983), changes in ionic composition could contribute to different patterns of motility, ultimately affecting fertilization success. If sperm motility is impaired in low-salinity environments due to local adaptation to brackish conditions (Nilsson *et al*., 2014), this may limit the effectiveness of recent habitat restoration efforts targeting freshwater systems aimed at reversing recruitment failures.

In this study, we investigate how sperm velocity in coastally collected pikes varies in deionized and local brackish water and over time following activation. Specifically, we test the following hypotheses: (i) sperm velocity is higher in local brackish water than in deionized water, reflecting local adaptation to brackish spawning conditions; and (ii) if coastal populations exhibit reduced intrinsic sperm quality, overall sperm velocity will be low across both activation media, indicating that reproductive impairment is not solely driven by environmental salinity conditions. By disentangling environmental effects (activation medium) from intrinsic sperm performance, we aim to determine whether reduced fertilization potential in Baltic coastal pike populations is primarily constrained by local environmental conditions, compromised male reproductive quality, or an interaction between the two. Our findings have implications for conservation strategies and habitat restoration efforts targeting declining coastal populations in the Baltic Sea.

## Materials and Methods

### Fish sampling

This study was conducted at two locations along the Swedish Baltic Sea coastline, a region characterized by salinity level of 4.5–8.0 ppt and a geomorphologically variable coastline with archipelagos that provide many shallow inlets and bays, offering sheltered spawning habitats for many Baltic fish species. Baltic-resident pike use such areas for spawning around March–May (Arlinghaus *et al*., 2023), while anadromous pike instead migrate to rivers or coastal freshwater wetlands to spawn.

On 3 May 2024, 12 adult, ready-to-spawn male pike were caught using fyke nets in brackish water spawning grounds within the Sankt Anna Archipelago and kept in holding nets near Stora Rimmö (Fig. 1) until sperm collection. On 24 March 2025, six additional adult males were caught at the outlet of a creek in Hanöbukten bay and kept under similar conditions near Åhus (Fig. 1). Based on these sampling locations, the 2024 individuals were likely brackish-spawning residents, whereas the 2025 individuals were likely anadromous pike entering freshwater to spawn.

Prior to sperm collection, all individuals were anesthetized in a 100-l plastic container filled with locally sourced brackish water with the addition of benzocain hypochlorite solution to a final concentration of 200 mg/l. Following ataxia (i.e. slowed respiration by observation of the movement of the operculum and loss of the righting reflex), sperm were extracted by applying gentle hand pressure to the posterior part of the abdomen. To avoid contamination and activation of sperm during stripping, the area around the genital pore was dried carefully prior to milt collection. Sperm was collected into 1-ml syringes, capped and stored in an ice box (3–4°C) for no longer than 1 h until sperm analysis. Samples were not transported between sites; sperm analyses were conducted on-site immediately after collection to minimize storage time and potential handling effects. After sperm collection, we measured the total length (cm) of each male (Table S5). The average total length was 52.3 cm (±SD, 5.8 cm) in males from Stora Rimmö and 51 cm (±8.4 cm) in males from Åhus. All individuals were allowed to recover in fresh brackish water inside a carp sack (a floating recovery bag commonly used in fisheries to temporarily hold live fish) and were returned to their natural environment.

### Sperm motility evaluation

Sperm samples were activated using two solutions: (i) deionized water (0 ppt) and (ii) locally collected brackish water (6 or 7 ppt). In 2024, brackish water was obtained from Stora Rimmö (6 ppt), while in 2025 it was collected from ^°^Åhus (7 ppt). For each individual, sperm was activated separately in both solutions. To initiate activation, a drop of semen was added to 200 μl of the respective activation solution. Due to the high viscosity of semen, precise volume measurements were not possible (Magnotti *et al*., 2018). Subsequently, 3 μl of the resulting mixture was quickly transferred to a disposable Leja chamber slide with a depth of 20 μl (IMV Technologies, L’Aigle, France) for analysis. Each activation treatment was replicated twice per individual. The temperature of the activation medium was recorded at the time of analysis and closely matched natural spawning conditions (3–4°C).

Sperm velocity (μm s^*−*1^) after activation was calculated using a portable microscope and a computer-assisted sperm analysis (CASA) software (UB 200i Series Microscope and C13-ON camera, PROiSER R + D). CASA settings (frame rate: 60 fps; particle size thresholds: 1–50 μm^2^) were kept constant across all samples and years. Measurements were assessed at three post-activation time intervals (10–15, 25–30 and 40–45 s) as pike sperm have a very short duration of motility, up to a maximum of 90 s in fresh water at 4°C (Babiak *et al*., 1995, 1997; Hulak *et al*., 2008*b*, 2008*a*).

We focused on three commonly used and correlated sperm velocity parameters returned by CASA; curvilinear velocity (VCL), straight-line velocity (VSL) and average path velocity (VAP). These metrics were chosen because they are well-established predictors of fertilization success in externally fertilizing fish (Gage *et al*., 2004; Liljedal *et al*., 2008; Skjæraasen *et al*., 2009). CASA occasionally detects non-motile sperm that appear to move due to fluid flow (i.e. floating cells). These cells were identified as those with an absolute difference between VCL and VSL values ≤10 μm s^*−*1^. Such cells were reclassified as static, and their velocity values were set to zero across all metrics. This correction enabled the analyses to retain and compare velocity estimates across all time points, including 40–45 s post-activation, when sperm immobility was common, while avoiding artificial inflation of velocity values due to flow-induced movement. For each individual, activating solution and time interval, mean velocity values were calculated across all sperm cells. For each sample, a minimum of 200 motile sperm cells were tracked across the time interval. To summarize variation among the three correlated sperm velocity parameters, we also performed a principal component analysis (PCA) using the prcomp function in base R. Each metric was standardized (mean = 0, SD = 1) prior to analysis. The first principal component (PC1), which explained 97.2% of the total variance, was retained for further analysis (Table S1). All three velocity measures contributed approximately equally to PC1. To facilitate interpretation, PC1 scores were multiplied by −1, as the original loadings were negative, allowing higher PC1 values to correspond to higher sperm velocity. In addition to sperm velocity, we quantified the proportion of motile sperm (% motility) for each individual, activating solution and time interval, to distinguish overall sperm activation (motility) from swimming performance (velocity).

In 2025, we also collected water from the river Helgeå, near Åhus, to compare potential differences with the deionized water used. Linear mixed-effects models (LMMs; see Table S6) were applied to check for any effects of treatment (freshwater vs deionized) and post-activation time on sperm velocity metrics (VCL, VSL, VAP, PC1) and sperm motility (%). No significant effect of treatment was detected (all *P >* 0.45), confirming that deionized water was suitable as the activating medium in all assays.

### Statistical analysis

LMMs were performed to assess the effects of activation solution, post-activation timing, location/year (hereafter, location) and their interactions on each velocity parameter (VCL, VSL and VAP, see Fig. S1, Tables S3 and S4), the first principal component (PC1), as well as the proportion of motile sperm (%) (see Table S2). In all models, individual identity was included as a random effect to account for repeated measures. As predictors had more than two categories and interaction effects were of interest, Type III analysis of variance (ANOVA) (*Anova* function from the car package) was applied to the fitted LMMs to test the significance of fixed effects. When significant effects were detected, pairwise comparisons were performed using Tukey *post hoc* tests implemented in the emmeans package (Lenth and Piaskowski, 2025). Effect sizes are presented as estimated marginal mean differences with associated standard errors.

All statistical analyses were performed in *R* version 4.3.1. We constructed linear LMMs using the lme4 package (Bates *et al*., 2015). The assumptions of normality and homoscedasticity required for LMMs were checked for all models.

### Ethical statement

Fish collection and analysis were performed in accordance with Swedish legislation, under ethical permits number 01871-2025 and SU FV-1141-25.

## Results

### Sperm velocity

**Figure 2:**
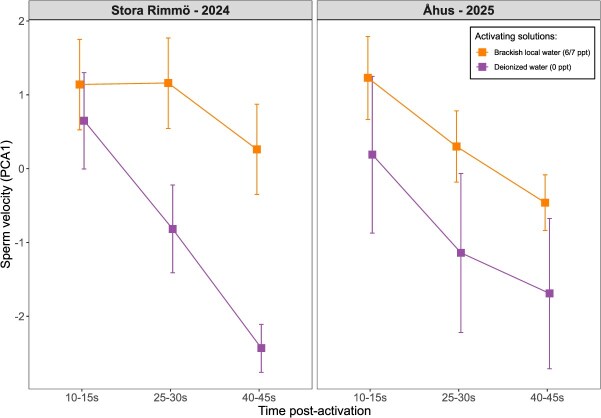
Effects of activating solution on sperm velocity (PC1) at 10–15, 25–30 and 40–45 s post-activation in Stora Rimmö–2024 (left panel) and in Åhus–2025 (right panel). The treatments included brackish local water (6 or 7 ppt, orange) and deionized water (0 ppt, purple). The square dots indicate the means and the vertical whiskers indicate ±95% intervals. For significance of the contrasts, please refer to Table 1.

Sperm velocity (PC1) was significantly influenced by the three-way interaction between activating solution, time post-activation and location (LMM, *χ*^2^ = 12*.*36, *P* = 0.002; Table S2). The interaction between activating solution and time post-activation was also significant (*χ*^2^ = 95*.*30, *P <* 0.001, Fig. 2), indicating that treatment effects varied over time and between locations. Although the main effects of activating solution (*χ*^2^ = 1*.*62, *P* = 0.20) and location (*χ*^2^ = 0*.*03, *P* = 0.85) were not significant, sperm velocity declined significantly over time post-activation (*χ*^2^ = 34*.*08, *P <* 0.001). At 10–15 s post-activation, sperm velocities did not differ between activating solutions (*P >* 0.05, Table 1). At 25–30 and 40–45 s, sperm activated in brackish water swam significantly faster than those in deionized water (*P <* 0.001 in Stora Rimmö; *P* = 0.011 and 0.001 in Åhus, respectively) (Fig. 2). Overall, sperm in brackish water maintained higher velocities across both locations, with slightly weaker differences observed in Åhus.

**Table 1:** Tukey-adjusted pairwise comparisons of activating solutions (brackish vs deionized water) for sperm velocity (PC1) and motility (%) across locations and post-activation times

**Location**	**Time (s)**	**Comparison**	**Estimate**	**SE**	*t*	***P*-values**
**Sperm velocity (PC1)**						
	10–15	Brackish–deionized	0.49	0.39	1.27	0.207
Stora Rimmö–2024	25–30	Brackish–deionized	1.96	0.39	5.06	*<* 0.001(^***^)
	40–45	Brackish–deionized	2.78	0.40	7.04	*<* 0.001(^***^)
	10–15	Brackish–deionized	1.04	0.55	1.88	0.063
Åhus–2025	25–30	Brackish–deionized	1.44	0.55	2.62	0.010(^*^)
	40–45	Brackish–deionized	1.94	0.57	3.40	0.001(^**^)
**Sperm motility (%)**						
	10–15	Brackish–deionized	−0.48	5.75	−0.08	0.93
Stora Rimmö–2024	25–30	Brackish–deionized	9.46	5.79	1.63	0.11
	40–45	Brackish–deionized	30.92	5.91	5.23	*<* 0.001(^***^)
	10–15	Brackish–deionized	9.88	8.22	1.20	0.23
Åhus–2025	25–30	Brackish–deionized	15.94	8.22	1.94	0.056
	40–45	Brackish–deionized	23.23	8.57	2.71	0.008(^**^)

Estimates represent mean differences (Brackish*−*deionized) from the emmeans model outputs, with corresponding SE, *t*-ratio, and adjusted *P*-values. Significant values are shown in bold.

### Sperm motility

The percentage of motile sperm was also significantly influenced by the three-way interaction between activating solution, time post-activation and location (LMM, *χ*^2^ = 7*.*05, *P* = 0.029; Table S1). A two-way interaction between activating solution and time post-activation was also detected (*χ*^2^ = 65*.*04, *P <* 0.001), whereas the main effects of activating solution, time and location were not significant (*P >* 0.26 for all).

**Figure 3:**
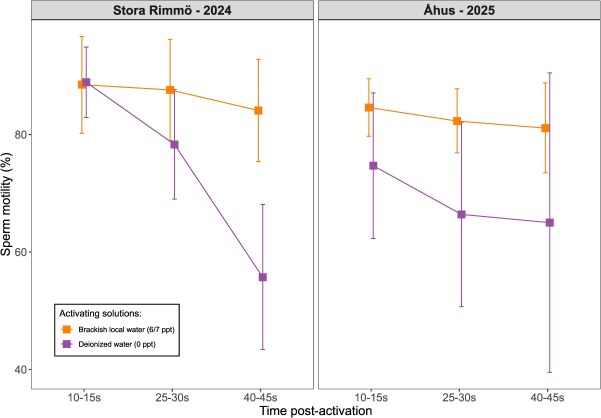
Effects of activating solution on sperm motility (%) at 10–15, 25–30 and 40–45 s post-activation. The treatments included brackish local water (6 or 7 ppt, orange) and deionized water (0 ppt, purple). The square dots indicate the means and the vertical whiskers indicate ±95% intervals. For significance of the contrasts, please refer to Table 1.

At 10–15 s post-activation, motility levels were high across treatments and years (mean range: 74.7–88.9%), with no significant differences between activating solutions (*P >* 0.23, Table 1). At 25–30 s, sperm in brackish water tended to remain more motile than those in deionized water, though differences were only marginal in ^°^Åhus (*P* = 0.056) and non-significant in Stora Rimmö (*P* = 0.11). By 40–45 s, motility had declined across treatments, but sperm activated in brackish water maintained significantly higher motility than those in deionized water (*P <* 0.001 in Stora Rimmö; *P* = 0.008 in Åhus, Fig. 3).

Overall, sperm in brackish water remained more motile at later times post-activation across both locations, although the magnitude of this difference varied slightly between locations.

## Discussion

Our study aimed to evaluate whether reduced sperm quality and/or salinity-dependent environmental effects could help explain the widespread recruitment failures observed in coastal pike populations in the Baltic Sea. By quantifying sperm velocity and motility between two salinity treatments, two locations/years and over the brief window in which fertilization occurs (Linhart *et al*., 2008), we found little evidence for intrinsic sperm quality issues. Instead, our results suggest that (i) sperm from coastal males remain functional in their local salinity, and (ii) time-dependent performance loss in deionized water occurs mainly after the critical fertilization window. Together, these findings suggest that reproductive limitations are more likely linked to later developmental stages, including potential maternal or early-embryo effects, rather than to male gamete function.

Across both sampling locations, sperm velocity generally declined rapidly with time post-activation, while motility declined rapidly in deionized water but not in brackish water. Differences in velocities between brackish and deionized water were negligible during the first 10–15 s after activation, which corresponds to the critical fertilization window in pike (typically *<*20 s; Linhart *et al*., 2008). During this period, sperm performance was high: curvilinear velocities in brackish water ranged from ~160–190 μm s^*−*1^, and 75–90% of sperm were motile immediately after activation. These levels match or exceed those reported for freshwater-spawning pike (Babiak *et al*., 1997; Hulak *et al*., 2008*b*), and fall within the range typically associated with high fertilization success in externally fertilizing fishes (Gage *et al*., 2004; Liljedal *et al*., 2008), indicating that intrinsic sperm quality is unlikely to limit fertilization under natural conditions. At later time points (25–30 and 40–45 s post-activation), however, sperm activated in brackish water consistently maintained higher velocities and motilities than those in deionized water, although the magnitude of these differences varied slightly between locations/years, with weaker effects observed in Åhus (2025). This weaker signal in Åhus (2025) may partly reflect the smaller sample size, which could increase variability and reduce the strength of detected interactions. Alternatively, it may also reflect biological differences between the study groups: fish sampled in 2025 (Åhus) were likely anadromous spawners entering fresh water to reproduce, whereas those collected in Stora Rimmö (2024) likely spawned in brackish spawning grounds. Greater freshwater exposure in anadromous spawners may produce different salinity tolerances, thereby reducing the contrast between salinity treatments.

Many freshwater and euryhaline fish species show improved sperm velocity and/or longevity in slightly saline or ion-enriched media compared with pure freshwater or deionized conditions (Rieniets and Millard, 1987; Alavi and Cosson, 2006; Linhart *et al*., 2008). For example, in brook trout (*Salvelinus fontinalis*), sperm motility was highest at moderate salinities (5 ppt) and declined in both lower and higher salinities (Bonislawska *et al*., 2015). Thus, the superior performance we observed in 6–7 ppt water is consistent with known physiological responses of freshwater sperm to moderate osmotic and ionic environments, which can stabilize membranes and prolong flagellar activity. Overall, the observed pattern suggests that osmotic stress in fresh water primarily reduces sperm longevity rather than immediate fertilization capacity, and would only affect reproductive success if fertilization was delayed beyond the normal temporal window. Together with the absolute velocity and motility values, these temporal dynamics demonstrate that coastal pike sperm remain functional across different salinity regimes, and that any performance loss in deionized water occurs mainly after the critical fertilization window. This suggests that migration to freshwater spawning habitats would not substantially impair fertilization capacity.

Several caveats warrant consideration. First, anecdotal reports from pike farmers of reduced milt volumes align with our own observations of relatively low sperm quantities (mean *±* SE: 0.45 ml *±* 0.06). Although sperm quality appears uncompromised, lower sperm quantities could contribute to decreased fertilization efficiency in natural populations, especially in flowing or wave-exposed environments. For example, in the green sea urchin *(Strongylocentrotus droebachiensis*), fertilization decreased with increasing water flow speeds, with the percentage of fertilized eggs dropping to levels <10% (Kregting *et al*., 2013). Second, our assays were conducted at 3–4°C; because sperm motility declines faster at warmer activation temperatures (Alavi and Cosson, 2006; Merino *et al*., 2023), the 6–8°C conditions typical of the late spawning seasons may amplify salinity-related effects. Third, our study examined sperm motility and velocity, without incorporating female reproductive traits or fertilization outcomes. In many externally fertilizing fish, sperm motility is not the sole determinant of fertilization and hatching success; egg quality, female–male compatibility, and post-fertilization development can also be limiting factors (Gage *et al*., 2004; Liljedal *et al*., 2008). In the freshwater spawning sea trout (*Salmo trutta*), e.g. fertilization proceeds in brackish water, but subsequent embryogenesis is impaired unless eggs are transferred to fresh water within hours (Rubin, 1994; Landergren and Vallin, 1998). In pike, salinity effects on early development appear to be strongly population-specific. While some studies on central and northern Baltic populations report successful hatching in both brackish and freshwater conditions (Bylund *et al*., 2001; Westin and Limburg, 2002), more recent work on a southern Baltic pike population documented complete absence of hatching at 0 psu (Jørgensen *et al*., 2010). Thus, even among coastal pike populations, tolerance to freshwater during embryogenesis varies, possibly reflecting differences between anadromous and brackish-spawning life-history strategies. The extent to which salinity affects fertilization and early development remain to be tested experimentally for our studied populations. Finally, environmental contaminants present in some Baltic spawning habitats, such as naturally produced brominated algal metabolites (OH-BDEs, Dahlgren *et al*., 2015), may also influence sperm energetics (van Boxtel *et al*., 2008; Legradi *et al*., 2014) or early developmental success (Kammann *et al*., 2006; Haldén *et al*., 2010) and represent and warrant future investigation.

In conclusion, our results indicate that sperm motility and velocity in coastal pike are not limiting during the fertilization window, and that observed declines in reproductive success are more likely attributable to factors beyond sperm quality. Future research should examine later life-history stages, such as fertilization and early embryonic development, as well as potential environmental influences, to clarify the mechanisms driving recruitment failures and guide conservation efforts for declining coastal Baltic pike populations.

## Supplementary Material

Web_Material_coag022

## Data Availability

Data and scripts are publicly available on Zenodo: https://doi.org/10.5281/zenodo.19000540
