# Supplementary material for: Sperm performance of coastal northern pike (Esox lucius L.) from the Baltic Sea shows no impairment between freshwater and brackish conditions
Source: Conserv Physiol. 2026 Apr 16;14(1):coag022. doi: 10.1093/conphys/coag022 (PMC13089564; doi:10.1093/conphys/coag022)
Supplement: Web_Material_coag022 [file web_material_coag022.zip › Supplementary materials.pdf]

Supplementary Material - Sperm performance of coastal northern pike (*Esox lucius L.*) from the Baltic Sea shows no impairment between freshwater and brackish conditions

Table S1: Loading values for sperm velocity principal components (PCs). Only PC1 was considered as this PC had a standard deviation $\geq$ 1. The proportion of variance and cumulative proportion of variance are presented for each PC.

**Loading (trait)**	**PC1**	**PC2**	**PC3**
VCL	0.57	-0.77	0.28
VSL	0.58	0.62	0.53
VAP	0.58	0.14	-0.80
**Standard deviation**	1.71	0.28	0.08
**Proportion of Variance**	0.972	0.026	0.002
**Cumulative Proportion**	0.972	0.998	1.000

Table S2: Effect of activating solution, post-activation time, and location on sperm velocity (PC1) and motility (%).

Response variable	N	Predictors	χ^2	*P*-values
**(a) Sperm velocity**				
		Activating solution	1.62	0.203
		Time post-activation	34.08	**<0.001(***)**
		Location	0.03	0.853
PC1 score	248	Activating solution * Time	95.30	**<0.001(***)**
		Activating solution * Location	0.66	0.416
		Time * Location	12.07	**0.002(**)**
		Activating solution * Time * Location	12.36	**0.002(**)**
**(b) Sperm motility**				
		Activating solution	0.01	0.933
		Time post-activation	2.65	0.266
		Location	0.30	0.586
% Motile sperm	248	Activating solution * Time	65.04	**<0.001(***)**
		Activating solution * Location	1.07	0.302
		Time * Location	0.38	0.825
		Activating solution * Time * Location	7.05	**0.029(*)**

Outputs of linear mixed models (LMMs) investigating the effects of activating solution, post-activation time, and sampling location on (a) sperm velocity (PC1) and (b) sperm motility (%). The total number of trials (N) is presented for each model, along with the Wald χ^2 statistic and corresponding *P*-value for each effect. χ^2 values were obtained using type III tests from the `car` package. Significant *P*-values are shown in bold.

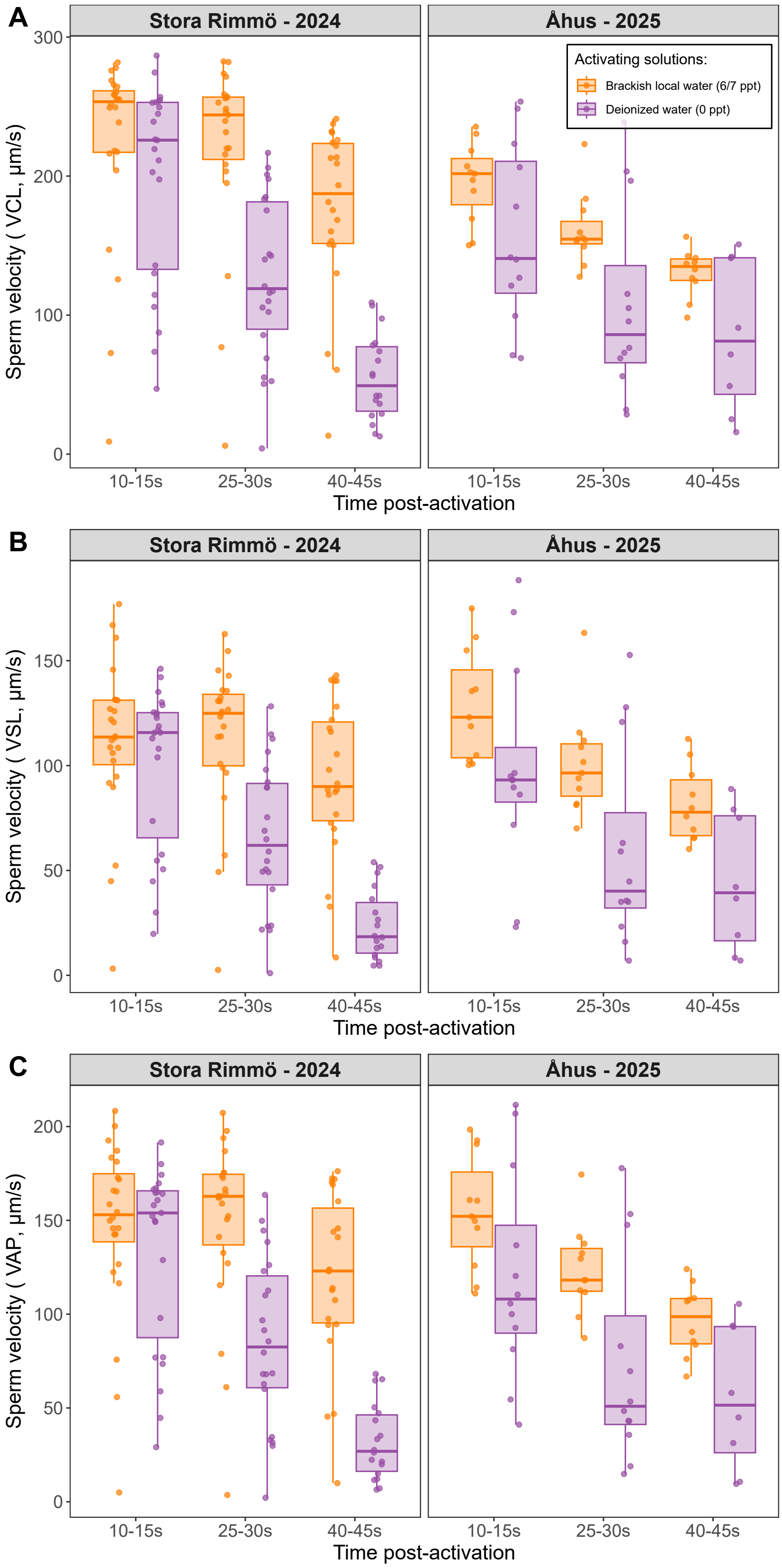

Fig. S1: Effects of activating solution on each velocity metrics (A: VCL, B: VSL, and C: VAP) at 10–15, 25–30, and 40–45 seconds post-activation. The treatments included brackish local water (6/7 ppt, orange) and deionized water (0 ppt, purple). In the box plots, the horizontal lines indicate the median (50th percentile) and quartiles (25th and 75th percentiles), and the vertical whiskers indicate the range of data within 1.5 times the interquartile range from the quartiles.

Table S3: Effect of activating solution, post-activation time, and location on sperm velocity metrics (VCL, VSL, and VAP).

Response variable	N	Predictors	χ^2	***P*-values**
**(a) VCL (Curvilinear velocity)**				
		Activating solution	2.41	0.12
		Time post-activation	46.64	**<0.001(***)**
		Location	1.76	0.18
VCL	248	Activating solution * Time	85.90	**<0.001(***)**
		Activating solution * Location	0.18	0.67
		Time * Location	6.07	**0.048(*)**
		Activating solution * Time * Location	14.53	**0.001(**)**
**(b) VSL (Straight-line velocity)**				
		Activating solution	1.22	0.27
		Time post-activation	19.86	**<0.001(***)**
		Location	1.62	0.20
VSL	248	Activating solution * Time	84.31	**<0.001(***)**
		Activating solution * Location	1.01	0.31
		Time * Location	16.32	**<0.001(***)**
		Activating solution * Time * Location	8.03	**0.018(*)**
**(c) VAP (Average path velocity)**				
		Activating solution	1.23	0.27
		Time post-activation	32.24	**<0.001(***)**
		Location	0.26	0.61
VAP	248	Activating solution * Time	97.57	**<0.001(***)**
		Activating solution * Location	0.89	0.35
		Time * Location	14.12	**<0.001(***)**
		Activating solution * Time * Location	12.78	**0.002(**)**

Outputs of linear mixed models (LMMs) investigating the effects of activating solution, post-activation time, and sampling location on sperm velocity metrics: (a) VCL, (b) VSL, and (c) VAP. The total number of trials (N) is shown for each model. Wald χ^2 values were obtained using type III tests from the `car` package. Significant *P*-values are shown in bold.

Table S4: Tukey-adjusted pairwise comparisons of activating solutions (brackish vs deionized water) for sperm velocity metrics (VCL, VSL, and VAP) across locations and post-activation times.

**Year**	**Time (s)**	**Comparison**	**Estimate**	**SE**	t	***P*-value**
**(a) Curvilinear velocity (VCL)**						
Stora Rimmö–2024	10–15	Brackish – Deionized	26.5	17.0	1.55	0.124
	25–30	Brackish – Deionized	95.1	17.2	5.54	**<0.001(***)**
	40–45	Brackish – Deionized	125.9	17.5	7.20	**<0.001(***)**
Åhus–2025	10–15	Brackish – Deionized	39.2	24.4	1.61	0.112
	25–30	Brackish – Deionized	53.6	24.4	2.20	**0.031(*)**
	40–45	Brackish – Deionized	69.3	25.3	2.74	**0.007(**)**
**(b) Straight-line velocity (VSL)**						
Stora Rimmö–2024	10–15	Brackish – Deionized	11.6	10.5	1.10	0.273
	25–30	Brackish – Deionized	47.2	10.6	4.45	**<0.001(***)**
	40–45	Brackish – Deionized	70.7	10.8	6.55	**<0.001(***)**
Åhus–2025	10–15	Brackish – Deionized	30.1	15.1	2.00	**0.049(*)**
	25–30	Brackish – Deionized	41.3	15.1	2.74	**0.008(**)**
	40–45	Brackish – Deionized	59.6	15.6	3.82	**0.0002(***)**
**(c) Average path velocity (VAP)**						
Stora Rimmö–2024	10–15	Brackish – Deionized	14.0	12.6	1.11	0.271
	25–30	Brackish – Deionized	62.7	12.7	4.94	**<0.001(***)**
	40–45	Brackish – Deionized	90.1	12.9	6.98	**<0.001(***)**
Åhus–2025	10–15	Brackish – Deionized	34.7	18.0	1.92	0.058
	25–30	Brackish – Deionized	49.7	18.0	2.76	**0.007(**)**
	40–45	Brackish – Deionized	63.3	18.7	3.39	**0.001(**)**

Tukey-adjusted pairwise contrasts comparing sperm velocities between activating solutions (brackish local water vs deionized water) for each location and post-activation time. Estimates represent mean differences (Brackish − Deionized) with standard error (SE), t-ratio, and adjusted P-values from the `emmeans` output. Significant values are shown in bold.

Table S5: Individual information

Origin	Sampling time	Males	Total length (cm)	Sperm volume (ml)
Stora Rimmö–2024	*3 May 2024*	m1	63	0.7
		m2	53	0.7
		m3	46	0.6
		m4	51.5	0.1
		m5	53	0.6
		m6	46	0.1
		m7	56	0.2
		m8	62	1
		m9	49.5	0.5
		m10	46	0.1
		m11	54	0.3
		m12	48	0.7
Åhus–2025	*24 March 2025*	m13	56	0.4
		m14	49	0.3
		m15	35	0.1
		m16	56	0.5
		m17	53	0.2
		m18	57	0.3

Table S6: Effect of treatment (freshwater vs deionized) and post-activation time on sperm velocity metrics (VCL, VSL, VAP and PC1) and sperm motility (%).

**Response variable**	**N**	**Predictors**	χ^2	***P*-values**
**(a) VCL (Curvilinear velocity)**				
		Treatment	0.52	0.47
VCL	68	Time	56.90	<**0.001(***)**
		Treatment * Time	2.16	0.34
**(b) VSL (Straight-line velocity)**				
		Treatment	0.29	0.59
VSL	68	Time	77.08	<**0.001(***)**
		Treatment * Time	0.81	0.67
**(c) VAP (Average path velocity)**				
		Treatment	0.51	0.47
VAP	68	Time	75.34	<**0.001(***)**
		Treatment * Time	2.01	0.37
**(d) PC1 (first principal component)**				
		Treatment	0.44	0.51
PC1 score	68	Time	74.10	<**0.001(***)**
		Treatment * Time	1.58	0.45
**(e) Sperm motility**				
		Treatment	0.01	0.95
% Motile sperm	68	Time	3.70	0.16
		Treatment * Time	2.04	0.36

Outputs of LMMs testing the effects of activating solution (treatment: freshwater vs deionized) and post-activation time on sperm velocity metrics (a, b, c and d) and sperm motility (d). Wald χ^2 values are from Type III tests (`car` package). Significant *P*-values are shown in bold.
